# Vaccination Accelerates Liver-Intrinsic Expression of Megakaryocyte-Related Genes in Response to Blood-Stage Malaria

**DOI:** 10.3390/vaccines10020287

**Published:** 2022-02-14

**Authors:** Frank Wunderlich, Denis Delic, Daniela Gerovska, Marcos J. Araúzo-Bravo

**Affiliations:** 1 Department of Biology, Heinrich-Heine-University, 40225 Düsseldorf, Germany; frank.wunderlich@hhu.de; 2 Boehringer Ingelheim Pharma GmbH & Co. KG, 88400 Biberach, Germany; 3 Fifth Department of Medicine (Nephrology/Endocrinology/Rheumatology), University Medical Centre Mannheim, University of Heidelberg, 68167 Heidelberg, Germany; 4 Computational Biology and Systems Biomedicine, Biodonostia Health Research Institute, 20014 San Sebastian, Spain; daniela.gerovska@biodonostia.org; 5 IKERBASQUE, Basque Foundation for Science, 48009 Bilbao, Spain; 6 TransBioNet Thematic Network of Excellence for Transitional Bioinformatics, Barcelona Supercomputing Center, 08034 Barcelona, Spain

**Keywords:** extramedullary megakaryo-/thrombopoiesis, liver, gene expression, blood-stage malaria, *Plasmodium chabaudi*, protective vaccination

## Abstract

Erythropoiesis and megakaryo-/thrombopoiesis occur in the bone marrow proceeding from common, even bipotent, progenitor cells. Recently, we have shown that protective vaccination accelerates extramedullary hepatic erythroblastosis in response to blood-stage malaria of *Plasmodium chabaudi.* Here, we investigated whether protective vaccination also accelerates extramedullary hepatic megakaryo-/thrombopoiesis. Female Balb/c mice were twice vaccinated with a non-infectious vaccine before infecting with 10^6^ *P. chabaudi*-parasitized erythrocytes. Using gene expression microarrays and quantitative real-time PCR, transcripts of genes known to be expressed in the bone marrow by cells of the megakaryo-/thrombocytic lineage were compared in livers of vaccination-protected and unprotected mice on days 0, 1, 4, 8, and 11 *p.i.* Livers of vaccination-protected mice responded with expression of megakaryo-/thrombocytic genes faster to *P. chabaudi* than those of unvaccinated mice, evidenced at early patency on day 4 *p.i.*, when livers exhibited significantly higher levels of malaria-induced transcripts of the genes *Selp* and *Pdgfb* (*p*-values < 0.0001), *Gp5* (*p*-value < 0.001), and *Fli1*, *Runx1*, *Myb*, *Mpl*, *Gp1ba*, *Gp1bb*, *Gp6*, *Gp9*, *Pf4*, and *Clec1b* (*p*-values < 0.01). Together with additionally analyzed genes known to be related to megakaryopoiesis, our data suggest that protective vaccination accelerates liver-intrinsic megakaryo-/thrombopoiesis in response to blood-stage malaria that presumably contributes to vaccination-induced survival of otherwise lethal blood-stage malaria.

## 1. Introduction

Malaria is still a threat to human health in tropical countries. In 2019, there were an estimated 229 million cases and 409,000 deaths globally [1]. Morbidity and mortality of malaria are caused by the blood-stages of parasitic protozoans of the genus *Plasmodium*, which invade and multiply in host red blood cells. *P. falciparum* is by far the deadliest *Plasmodium* species, causing about 99% of global malaria-related deaths [2]. Currently, an anti-malaria vaccine with an efficacy of more than 50%, neither against *P.* *falciparum* nor any other human malaria species, is yet to be commercially available [3,4].

*P. falciparum* shares several characteristics with *P. chabaudi* in mice, which is therefore a convenient experimental system to study the host defense and its responses to vaccination against blood-stage malaria [5,6]. In this model, vaccination with a non-infectious vaccine, consisting of erythrocyte ghosts isolated from *P. chabaudi*-infected erythrocytes, induces a healing course of otherwise lethal primary infections and reduces peak parasitaemia by approximately 30% [7,8]. This vaccination-induced survival of mice is associated with dramatic changes in the responsiveness of the liver to blood-stage malaria, which represents, besides the spleen [9,10], a major organ of the host defense against malaria [11]. Indeed, the liver of vaccinated surviving mice responds to blood-stage malaria with attenuated inflammation, augmented uptake of injected particles, reprogrammed metabolic processes and gene expression [8], epigenetic modifications of promoters of protein-encoding genes [12], altered expressions of miRNAs [13] and lincRNAs [14,15], and reshaped responses of NK cells [16]. Most remarkably, vaccination-induced survival was associated with an acceleration of extramedullary erythroblastosis induced in the liver by blood-stage malaria [17].

According to the canonical model of haematopoiesis, erythropoiesis occurs in the bone marrow, just as megakaryopoiesis. Both are dependent on each other insofar as they follow a common differentiation path within haematopoiesis and arise from common progenitors. Both nucleated erythroblasts and polyploid megakaryocytes can even arise from common bipotent megakaryocyte-erythroid progenitor (MEP) cells in the bone marrow [18,19,20,21]. These MEPs originate from long-term haematopoietic stem cells (LT-HSC) via short-term (ST)-HSCs, different multipotent progenitor (MPP) cells, and common myeloid progenitor cells, and MEPs further differentiate to megakaryocyte progenitors (MKP) and to the final megakaryocytes (MKs). Recently, however, there is increasing information suggesting that the commitment of HSCs to the megakaryocytic lineage may be much more complex than previously thought [20,21]. Indeed, MKs may not only arise from uni-, bi-, and multi-potent progenitor (MPP) cells, but also even directly from HSCs [22,23,24,25]. Moreover, megakaryopoiesis, just as erythropoiesis, is not restricted to the bone marrow. Recent evidence indicates that diverse stress can also induce extramedullary megakaryopoiesis in other organs, e.g., in the lung [26,27].

MKs in the bone marrow are large, polyploid cells with a diameter ranging from 30 μm to 100 μm and an average polyploidy of 16N (range 4N–128N) [28]. There is evidence accumulating that MKs do not only manipulate the haematopoietic niche, but also are critically involved in innate and adaptive immunity. Indeed, they exhibit diverse surface molecules to sense inflammation [28], to present MHC class I and II antigens [29,30], and to release immunoregulatory cytokines and chemokines, even packaged in microparticles [28]; their surface CD40L activates B cells, T cells, and macrophages via their surface CD40 [31]; and MKs possess antiviral activity [32], but are also targets for viruses such as Dengue [33] and, presumably, SARS-CoV-2 viruses [34].

The main immune function of MKs is to produce platelets or thrombocytes. Accordingly, MKs display cytoplasmic finger-like protuberances, parts of which are released into the bloodstream as anuleate prothrombocytes where they mature to thrombocytes [20,28]. Though anucleate, thrombocytes contain a diverse set of RNA species such as mRNA, miRNA, lncRNA, circRNA, and even viral RNA [35,36,37]. Moreover, there is evidence that thrombocytes are effective killers of *Plasmodium*-infected erythrocytes [38,39]. This killing is apparently associated with a loss of circulating thrombocytes, which may explain why blood-stage malaria is often reported to be associated with development of thrombocytopenia, representing a severe complication in human malaria caused by both *P. falciparum* and *P. vivax* [40,41,42,43,44,45,46,47,48].

Recently, protective vaccination of mice against *P. chabaudi* blood-stage malaria was found not only to accelerate extramedullary erythroblastosis in the liver, but also to induce an up-regulation of mir-142-3p in the liver [13]. This miRNA-species was previously described to stringently control specific cytoskeletal rearrangements required for maturation and function of megakaryocytes, and its genetic deletion resulted in cytoskeletal dys-integrity, abnormal proplatelet formation, and thrombocytopenia [49]. We therefore suspected that blood-stage malaria does not only induce extramedullary erythroblastosis in the liver but possibly also promotes development of megakaryo- and thrombocytes, respectively, in the liver. Under identical experimental conditions as our previous studies on vaccination-accelerated malaria-induced extramedullary erythroblastosis in the liver [17], we addressed here whether extramedullary development of megakaryo-/thrombocytes can take place in the liver of mice infected with blood-stage malaria of *P. chabaudi* and, if so, whether this may even be affected by protective vaccination. We used gene expression microarray technology and quantitative RT-PCR to identify and validate malaria- and vaccination-responsive genes, known to be preferentially expressed in cells of the megakaryo-/thrombocytic lineage, in the liver of mice during the course of primary blood-stage infections of *P. chabaudi*, taking a healing outcome in vaccination-protected mice and a lethal outcome in non-vaccinated mice.

## 2. Material and Methods

### 2.1. Protective Vaccination of Mice against Blood-Stage Malaria

**Figure 1:**
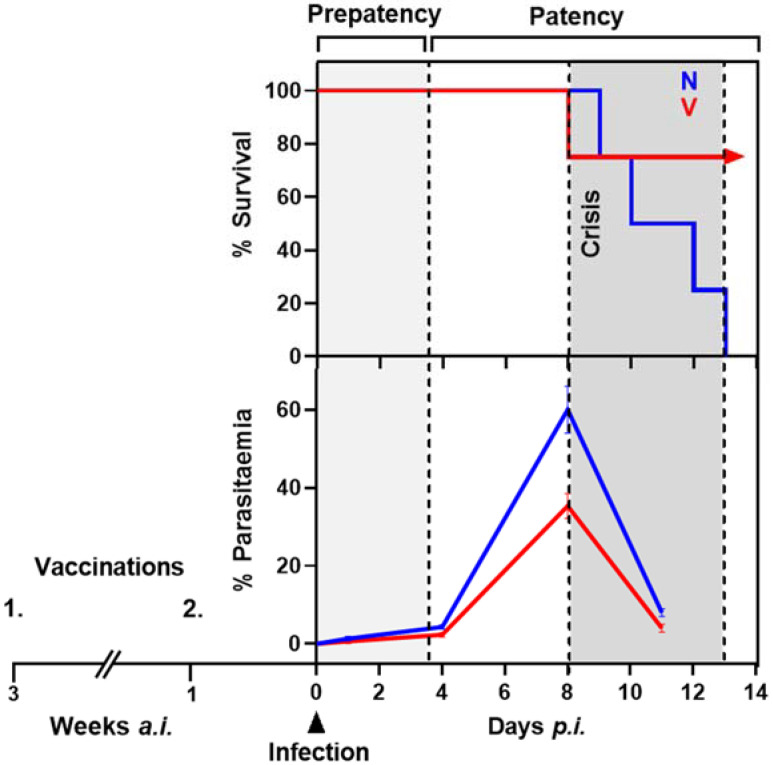
Scheme summarizing the effect of vaccination on outcome of blood-stage malaria with *P. chabaudi*. Female Balb/c mice were vaccinated with a non-infectious vaccine on weeks 3 and 1 *a.i.* (*ante infectionem*), before challenging vaccinated (V) and non-vaccinated (N) mice with 10^6^ *P. chabaudi*-infected erythrocytes on day 0. At early patency on day 4 *p.i.*, about 1–5% parasitized erythrocytes appear in peripheral blood without any difference between V and N. Peak parasitaemia occurs on about 8 *p.i.*, reaching about 60% in N and only about 40% in V. The crisis phase of infection is characterized by dramatically falling parasitaemias and the death of all N, whereas the majority of V survive the infections.

These experiments were previously performed as summarized elsewhere [16,17]. Female Balb/c mice aged 10–12 weeks, bred under specified pathogen-free conditions in the central animal facilities of the University of Düsseldorf, were maintained in our specific sterile lab facilities, where they received a standard diet (Wohrlin, Bad Salzuflen, Germany) and water ad libitum throughout the experiments. Mice were vaccinated with a non-infectious vaccine consisting of erythrocyte ghosts isolated from *P. chabaudi*-parasitized erythrocytes [50], as described previously [7,8]. These membrane ghosts are associated with parasite-synthesized proteins and presumably autoantigens [51,52]. Approximately 10^6^ ghosts were suspended in 100 µL Freund’s complete adjuvant (FCA) and subcutaneously injected twice at a 2-week interval, while control mice were in parallel treated only with FCA (Figure 1). The efficacy of protective vaccination depends on genes of the H-2 complex, the non-H-2 background, sex, and testosterone of mice as briefly summarized elsewhere [53]. The female Balb/c mice, used here, exhibit a ‘malaria-nonhealer’ non-H-2 background and a ‘malaria-healer’ H-2^d^ haplotype [53] and require the two-time vaccination to survive *P. chabaudi* blood-stage infections, otherwise taking a lethal outcome (Figure 1).

### 2.2. P. chabaudi Malaria and Liver Sampling

One week after the second vaccination, vaccinated (V) and non-vaccinated mice (N) were concomitantly infected with 10^6^ *P. chabaudi*-parasitized erythrocytes. These primary infections take a similar course in terms of parasitaemia in V- and N-mice (Figure 1). 

For liver sampling, groups of 3 animals from both V and N were sacrificed at different phases of infections: upon infection on day 0 *p.i.*, at early prepatency on day 1 *p.i.*, at early patency on day 4 *p.i.*, at peak parasitaemia on day 8 *p.i*., and towards the end of the crisis phase on day 11 *p.i.* Parasitaemias, previously determined in blood smears of sacrificed mice [13], corresponded to those determined in living infected mice under identical experimental conditions [8]. Four mice in both the V and N group were not sacrificed: all 4 mice in the N-group died during crisis, whereas 3 out of the 4 mice in the V-group survived the infection for at least 3 weeks, in accordance with our previous results [8].

### 2.3. Hybridization of Mouse Whole Genome Oligo Microarrays

The livers, isolated as described in Figure 1, were rapidly frozen in liquid nitrogen and stored at −80°C until use. Individual frozen livers were ‘pulverized’ under liquid nitrogen, and total RNA of individual livers was isolated by the standard Trizol method (Qiagen, Hilden, Germany) followed by cleaning with the miRNeasy Kit (Qiagen). Equivalents of 100 ng from each RNA sample were used to produce Cy3-labeled cRNA with the Agilent Low Input Quick Amp Labeling Kit (Agilent Technologies, Santa Clara, CA, USA) according to the manufacturer’s protocol. Agilent’s 8 × 60 K oligo microarrays (design number 028005) were then hybridized using the Agilent Gene Expression Hybridization Kit.

### 2.4. Analyses of Microarrays

Microarrays were scanned with the Agilent’s Microarray Scanner System (Agilent Technologies) using the Agilent Feature Extraction (FE) software to read out and process the microarray image files. The FE software determines feature intensities including background subtraction, rejects outliers, and calculates statistical confidences of feature intensities. All the 30 microarrays were normalized by the quantile method. Microarray data are available at both the EMBL-EBI Array Express repository (Array accession number: E-MTAB-4791) and the NCBI’s Gene Expression Omnibus (GEO) database with accession number GSE129133. Global transcriptomics analyses over all normalized 30 microarrays were previously performed including heat map of the most highly variable transcripts, hierarchical clustering dendrograms (calculated using the unweighted pair group method with arithmetic mean and Euclidean distance measure), and Principal Component Analysis (PCA), as previously detailed [14]. Gene expression was determined from the normalized microarrays prepared from the individual livers of both V-and N-mice mice infected with *P. chabaudi* on days 0, 1, 4, 8, and 11 *p.i.* Relative levels of gene expression were measured as light intensities above normalization level and given as means ± SD as dispersion metric in all figures. T-test was used to determine statistical significance of differences in gene expression levels between V- and N-mice both at a given day *p.i.* and during the intervals between different sampling days *p.i.*

### 2.5. Real-Time Quantitative PCR

To check the reliability of the microarray data, we analyzed the same biological samples, specifically equivalents of the frozen and ‘pulverized’ individual livers. The High-Capacity cDNA Reverse Transcription Kit (Thermo Fisher, Waltham, MA, USA) and specific TaqMan mRNA assays (Thermo Fisher) were used for reverse transcription of mRNAs produced by the following genes: *Thpo* (assay ID: Mm00437040_m1), *Stat3* (assay ID: Mm01219775_m1), *Etv6* (assay ID: Mm01261325_m1), *Myb* (assay ID: Mm00501741_m1), *Gp9* (assay ID: Mm00497671_g1), *Notch4* (assay ID: Mm00440525_m1), *Tubb1* (assay ID: Mm01239914_g1), and *Selp* (assay ID: Mm00441297_m1). PCR reactions were performed using the TaqMan^®^ gene expression master mix (Thermo Fisher) according to the manufacturer’s instructions on a 7900HT real-time PCR System, as previously described [17]. All samples were run in duplicate and raw C_t_ values were obtained from the SDS software v.2.4 and normalized C_t_ values were calculated using *Gapdh* (assay ID: Mm99999915_g1) as the housekeeping gene. The comparative C_t_ method (2^−ΔΔCt^) was used to calculate fold change of expression [54]. Statistical significances between V- and N-mice were analyzed with the two-tailed unpaired heteroskedastic Student’s t-test (* *p*-value <  0.05).

## 3. Results

### 3.1. Megakaryocyte-Related Genes of Vaccinated Mice Increase Their Average Expression Three Days Earlier Than Non-Vaccinated Mice

**Figure 2:**
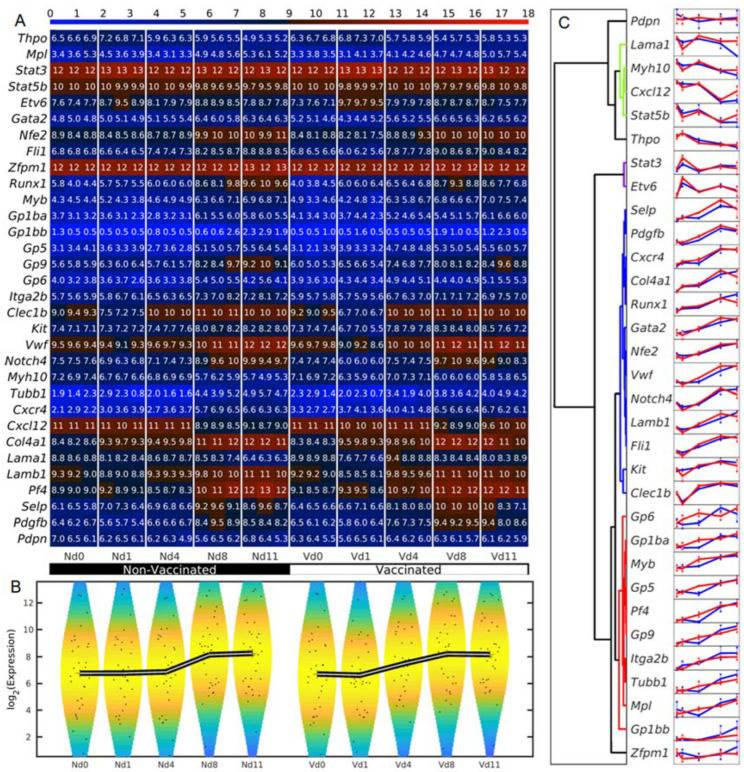
Dynamics of the expression of the megakaryocyte-related genes. (**A**) Heat map of the expression of megakaryocyte-related genes. The color bar codifies the genes expression in log2 scale. Higher gene expression corresponds to redder color. (**B**) Violin plots associated to the expression distribution of megakaryocyte-related genes. The black lines mark the trajectories of the position of the means of the megakaryocyte-related gene expression. The points represent the spread of the expression of the genes. (**C**) Hierarchical clustering performed using the correlation metric and the Ward linkage method of time series profiles. The blue and red profiles correspond to non-vaccinated and vaccinated samples, respectively.

The expression dynamics of megakaryocyte-related genes in response to blood-stage malaria were investigated in the liver of non-vaccinated and vaccination-protected mice. The numerical expression values of all the replicates of the megakaryocyte-related genes were compared and are shown in the heatmap in Figure 2A, revealing various dynamics patterns of the megakaryocyte-related genes. Some of the genes had similar profiles for vaccinated and non-vaccinated samples, while others had different profiles for the two sample groups. In general, the megakaryocyte-related genes increased their expression with the time. Vaccinated mice increased their average expression on day 1 *p.i.*, whereas non-vaccinated mice increased their average expression on day 4 *p.i.* (Figure 2B). To study the different typologies of expression profiles, we performed a hierarchical clustering of the expression dynamics (Figure 2C). We found four main types of trajectories: a few genes (from *Pdpn* to *Thpo*) followed constant or decreasing trajectories; *Stat3* and *Etv6* had a shot of expression on day 1 *p.i.*; a set from *Selp* to *Clec1b* presented strongly increasing trajectories; and another set from Gp6 to Gp1bb also presented increasing trajectories but with strong differences on certain days between vaccinated and unvaccinated samples. The detailed analysis of these trajectories and their differences between vaccinated and non-vaccinated samples is performed in the following sections.

### 3.2. Vaccination Accelerates Inverse Down- and Upregulation of Thpo and Mpl Expression in the Liver in Response to Blood-Stage Malaria

**Figure 3:**
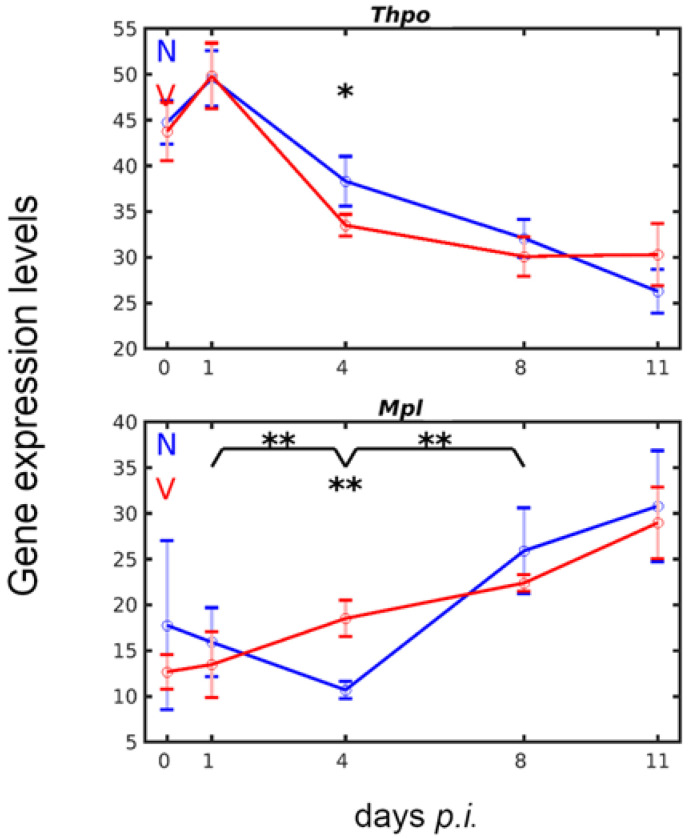
Expression courses of the thrombopoietin (TPO)-encoding *Thpo* gene and the TPO receptor- encoding *Mpl* in the liver of female Balb/c mice. RNA was isolated from individual livers prepared from vaccination-protected (V, red) and non-vaccinated mice (N, blue) infected with *P. chabaudi* blood-stage malaria on days 0, 1, 4, 8, and 11 *p.i.* Gene expression levels in linear scale are plotted as means of three microarrays ± SD. The number of ‘*’ marks over interval lines between sampling points and over the sampling points indicate statistical significance between two intervals and between a given sampling point, respectively, between vaccinated and non-vaccinated mice: * *p* < 0.05; ** *p* < 0.01.

Thrombopoietin (TPO) encoded by *Thpo* and its receptor MPL control megakaryo-/thrombopoiesis in the bone marrow [55,56]. *Thpo* is not only expressed in the bone marrow and in the kidney, but also mainly in the liver [55,56]. The liver of the female Balb/c mice, investigated here by gene expression microarrays, displayed a high constitutive *Thpo* expression of approximately 44 above normalization level on day 0 *p.i.* (Figure 3). Upon infection with blood-stage malaria, however, this level was slightly increased to approximately 49 at early prepatency on day 1 *p.i.*, before it steadily dropped to approximately 27 above normalization level towards the end of the crisis on day 11 *p.i.* Protective vaccination did not change the constitutive expression levels of *Thpo*, and it also did not affect the slight, but not significant, malaria-induced increase in expression during the first day of infection. However, the following decrease of *Thpo* transcripts from early prepatency on day 1 *p.i.* to early patency on day 4 *p.i.* was accelerated since the transcript levels of *Tpho* were significantly (*p*-value < 0.05) lower in the liver of vaccination-protected mice than in unprotected mice on day 4 *p.i.*

The time course of *Mpl* expression in response to blood-stage malaria totally differed from that of *Thpo* both in vaccination-protected and unprotected mice. The constitutive expression of *Mpl* on day 0 *p.i.* was about the same, i.e., about 15 above normalization level, in both vaccination-protected and unvaccinated mice, and this level is only one third of the constitutive expression level of *Thpo* (Figure 3). Infection of unvaccinated mice with *P. chabaudi* appeared to cause a slight decrease in *Mpl* transcripts on day 4 *p.i.*, before *Mpl* expression continuously increased, reaching its highest levels, 31, towards the end of the crisis phase on day 11 *p.i.* In contrast to unvaccinated mice, malaria infection caused an almost linear increase in hepatic *Mpl* expression of vaccinated mice on day 4 *p.i.*, before reaching about the same maximum level on day 11 *p.i.* as that of unvaccinated mice. It is therefore at early patency on day 4 *p.i.* that a significantly (*p*-value < 0.01) higher expression of *Mpl* occurred in the liver of vaccination-protected mice than in unvaccinated mice.

### 3.3. Vaccination Reshapes Malaria-Induced Hepatic Expression of Genes Encoding Transcription Regulators Involved in Megakaryopoiesis

**Figure 4:**
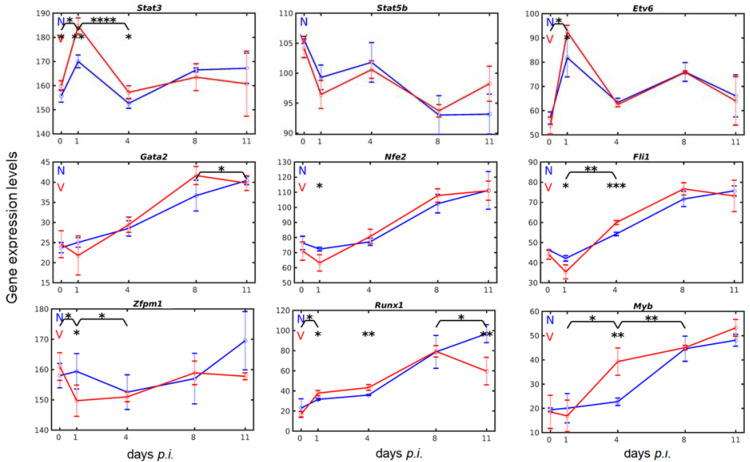
Expression trajectories of genes encoding transcription factors in the liver of vaccinated (V, red) and unvaccinated mice (N, blue). RNA was isolated from individual livers prepared from mice infected with *P. chabaudi* blood-stage malaria on different days *p.i.* Gene expression levels are plotted as means of three microarrays ± SD. The number of ‘*’ marks over interval lines between sampling points and over the sampling points indicate statistical significance between two intervals and between a given sampling point, respectively, between vaccinated and non-vaccinated mice: * *p* < 0.05; ** *p* < 0.01; *** *p* < 0.001; **** *p* < 0.0001.

TPO signaling through MPL requires the tyrosine kinase Janus kinase 2 (JAK2) and several other downstream signaling pathways including those mediated by signal transducers and activators of transcription (STAT)3 and STAT5 [20,57]. Figure 4 shows the expression courses of *Stat3* and *Stat5b* in the liver in response to blood-stage malaria. Remarkably, expression of *Stat3* and *Stat5b* were inversely responsive to malaria, but only *Stat3* expression significantly responded to vaccination. Thus, malaria induced a significantly (*p*-value < 0.01) higher expression of *Stat3* in vaccination-protected mice on day 1 *p.i.* than in unvaccinated mice, before expression highly significantly (*p*-value < 0.0001) dropped from about 185 to about 158 on day 4 *p.i.* and then remained about the same at peak parasitaemia and towards the end of the crisis phase. The constitutive expression of *Stat5b* on day 0 *p.i.* was lower than that of *Stat3*, and its time course of expression inversely progressed to that of *Stat3* in response to both malaria and vaccination.

Among the transcription factors known to be involved at different stages of megakaryopoiesis in the bone marrow [20,57], there are ETV6 (ETS, erythroblast transformation specific, variant 6), GATA2 (Gata binding protein 2), NF-E2 (nuclear factor, erythroid 2), FLI1 (friend leukemia integration 1 transcription factor), ZFPM1 (Zinc finger protein, multitype 1; FOG1), RUNX1 (runt-related transcription factor), and MYB (myeloblastosis oncogene). In the liver, the genes encoding these transcription factors were constitutively expressed at different levels on day 0 *p.i.*, and they were differently responsive to both malaria and protective vaccination (Figure 4).

*Etv6* encodes a transcriptional repressor belonging to the ETS family and is also known to be expressed in MEPs [20,57,58]. Remarkably, hepatic *Etv6* expression in response to both malaria and vaccination was very similar to that of *Stat3*, though the constitutive expression of *Etv6* in the liver was only about 1/3 of that of *Stat3*, with approximately 58 above normalization level (Figure 4). Infection with blood-stage malaria induced an increase in *Etv6* expression on day 1 *p.i.*, which was significantly (*p*-value < 0.05) higher in vaccination-protected than unvaccinated mice. Thereafter, *Etv6* expression dropped to approximately 64 on day 4 *p.i.* and then took about the same course in both vaccinated and unvaccinated mice. *Gata2* has been described to be expressed in MEPs and its overexpression promotes megakaryopoiesis at the expense of erythropoiesis in the bone marrow [20,59]. In the liver, the constitutive expression of *Gata2* was approximately 24 above normalization level in both unvaccinated and vaccinated mice (Figure 4). Infection with malaria induced an almost linear increase in *Gata2* expression in unvaccinated mice, which did not significantly differ from that in vaccinated mice. Only towards the end of the crisis phase on day 11 *p.i.*, there was a significant (*p*-value < 0.05) decrease in *Gata2* expression in vaccinated vs. unvaccinated mice. *Nfe2* is known to be expressed in MEPs and involved in the control of the terminal stage of megakaryocyte differentiation in the bone marrow, specifically in the megakaryo-specific TUBB1 proplatelet production [20,60]. The time course *Nfe2* expression in the liver of both unvaccinated and vaccinated mice occurred similarly to that of *Gata2* with the difference that *Nfe2* exhibited a much higher constitutive expression than *Gata2*, and *Nfe2* expression at early prepatency on day 1 *p.i.* was significantly (*p*-value < 0.05) lower in vaccinated mice than in unvaccinated mice (Figure 4). *Zfpm1* encoding the transcriptional factor FOG1 is essential for both erythroid and megakaryocytic differentiation. Indeed, FOG1 forms heterodimers with GATA2 and GATA1 in MEPs: GATA1/FOG1 promote erythropoiesis, whereas GATA2/FOG1 promote megakaryopoiesis at the expense of erythropoiesis [20]. It is therefore not surprising that the expression of *Zfpm1* exhibited by far the highest constitutive expression, approximately 158 above normalization level in the liver among the other transcription factors examined here (Figure 4). Upon infection with malaria, the expression of *Zfpm1* was only slightly—if at all—changed during progression of infection in unvaccinated mice; only towards the end of the crisis phase on day 11 *p.i.*, there was an increase in *Zfpm1* expression. In vaccinated mice, however, the liver responded to malaria with a significant (*p*-value < 0.05) decrease in *Zfpm1* expression from 162 to 150 on day 1 *p.i.*, before *Zfpm1* expression began to slightly increase, reaching approximately 159 above the normalization level at peak parasitaemia on day 8 *p.i.*, which is, however, apparently in the same range as the constitutive expression level on day 0 *p.i.*

*Fli1* is currently thought of being mainly expressed in the megakaryocyte lineage and acting as a suppressor of erythroid differentiation [20,57,61]. In the liver of unvaccinated mice, the constitutive expression of *Fli1* on day 1 *p.i.* was approximately 46 above the normalization level (Figure 4). Infection with malaria induced a slight decrease in *Fli1* expression at early prepatency on day 1 *p.i.*, before a continuous increase reaching a maximum on day 8 *p.i.* In vaccination-protected mice, however, the initially decreased expression of *Fli1* was significantly (*p*-value < 0.05) lower than that in unvaccinated mice. Moreover, the subsequent increase in *Fli1* expression was significantly (*p*-value < 0.001) higher on day 4 *p.i.* and the maximum was reached at peak parasitaemia on day 8 *p.i.* in comparison with unvaccinated mice. Like *Fli1*, *Runx1* is known to be expressed mainly in the megakaryocyte lineage: it promotes proliferation of megakaryocyte progenitors, downregulates terminal differentiation of megakaryocytes, and represses the erythroid gene expression program [57,62,63]. In unvaccinated mice, the liver responded with an initially slight increase in *Runx1* expression from 22 to about 31 above normalization, which then remained at this level until early patency on day 4 *p.i.*, before a steady increase, reaching its maximum on day 11 *p.i.* (Figure 4). In vaccination-protected mice, however, the response to malaria of *Runx1* expression was significantly (*p*-value < 0.05) increased from approximately 18 on day 0 *p.i.* to approximately 39 on day 1 *p.i.*, and highly significantly (*p*-value < 0.01) to approximately 42 on day 4 *p.i.* Maximum expression occurred at peak parasitaemia, before a highly significant (*p*-value < 0.01) decline towards the end of crisis on day 11 *p.i.* as compared with unvaccinated mice. The *Myb*-encoded transcriptional activator is known to play an important role in expansion of hematopoietic progenitor cells. In general, *Myb* is described to be exclusively expressed in the erythrocyte lineage, but mutations in *Myb* were reported to skew differentiation commitment of progenitor cells in favor of excess megakaryopoiesis [64]. Moreover, *Myb* is expressed in megakaryocyte progenitors expanding in vitro in mouse bone marrow or fetal liver cell preparations [65]. In the liver of unvaccinated mice, the constitutive expression of *Myb* was among the lowest of all transcription factor-encoding genes investigated here (Figure 4). In response to malaria, its expression was delayed for at least 4 days before an increase was observed, reaching a maximum towards the end of the crisis phase on day 11 *p.i.* In vaccinated mice, however, there was also an initial impairment of *Myb* expression, but lasting only for 1 day. Subsequently, there was an increase in *Myb* expression on day 4 *p.i.*, which was significantly higher (*p*-value < 0.01) than in unvaccinated mice, whereas maximal expression of *Myb* occurred on day 11 *p.i.*, which was slightly higher than that in unvaccinated mice.

### 3.4. Vaccination Accelerates Malaria-Induced Expression of Genes Encoding Surface Proteins on Cells of the Megakaryocytic Lineage in the Liver

**Figure 5:**
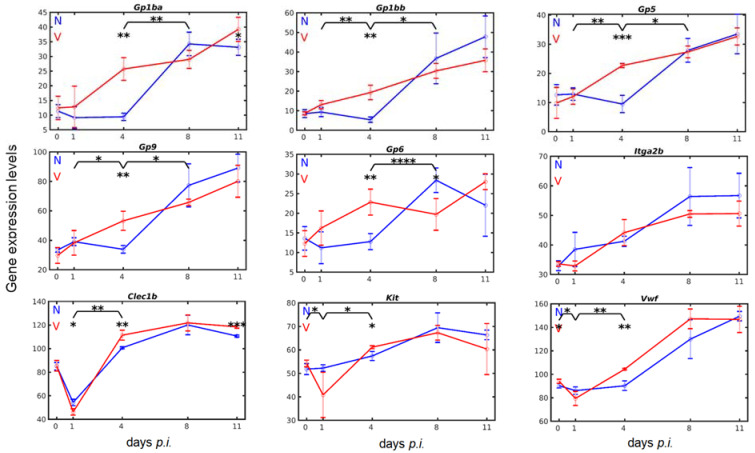
Time courses of hepatic expression of genes known to be expressed on the surface of cells of the megakaryo-/thrombocyte lineage. RNA was isolated from individual livers prepared from vaccination-protected (V, red) and non-vaccinated mice (N, blue) infected with *P. chabaudi* blood-stage malaria on days 0, 1, 4, 8, and 11 *p.i.* Gene expression levels in linear scale are plotted as means of three microarrays ± SD. The number of ‘*’ marks over interval lines between sampling points and over the sampling points indicate statistical significance between two intervals and between a given sampling point, respectively, between vaccinated and non-vaccinated mice: * *p* < 0.05; ** *p* < 0.01; *** *p* < 0.001; **** *p* < 0.0001.

CD42b encoded by *Gp1ba* (glycoprotein Ib alpha) is a characteristic surface protein on megakaryo-/thrombocytes, forming a disulfide-bonded heterodimer with the *Gp1bb* (glycoprotein Ib beta)-encoded GPIbb. This heterodimer is non-covalently complexed with GPIX (glycoprotein IX) encoded by *Gp9 20* and GPV (glycoprotein V) encoded by *Gp5*. The GPIB-V-IX complex is involved in different stages of megakaryocyte differentiation as well as in thrombocyte activation [20]. In the liver of mice, *Gp1ba*, *Gp1bb*, *and Gp5* exhibited similar low constitutive expression levels in the range of 9–12 above normalization; only *Gp9* was constitutively expressed at a higher level of approximately 32 (Figure 5). Expression time courses of these four genes were similar during primary malaria infections in unvaccinated mice insofar as there was an initial delay in response to blood-stage malaria during the first 4 days of infection, i.e., their transcript levels remained at about the same levels as the corresponding constitutive expressions. Only at early patency on day 4 *p.i.*, when parasitized erythrocytes began to appear in the peripheral blood, these transcript levels began to strongly increase, reaching maximal levels at peak parasitaemia with increasing tendency towards the end of the crisis phase on day 11 *p.i.* Remarkably, the liver of vaccination-protected mice did not display the initially delayed expressions of *Gp1ba*, *Gp1bb*, *Gp5*, and *Gp9* in response to blood-stage malaria, i.e., their mRNA levels were highly significantly (*p*-values < 0.01 and <0.001) increased at early patency on day 4 *p.i.* as compared with unvaccinated mice. Maximal levels were reached towards the end of the crisis phase on day 11 *p.i.* at about the same level as in unvaccinated mice; only *Gp1ba* was significantly higher expressed.

GPVI is another characteristic surface protein on megakaryo-/thrombocytes and serves as collagen receptor-mediating collagen-induced adhesion and for the activation of thrombocytes [20,66,67]. In the liver of both vaccinated and non-vaccinated mice, the expression course of *Gp6* in response to blood-stage malaria resembled that of *Gp1ba*, *Gp1bb*, *Gp5*, and *Gp9*, in particular with respect to a significantly (*p*-value < 0.01) higher expression in vaccinated mice on day 4 *p.i.* than in unvaccinated mice (Figure 5). The only highly significant (*p*-value < 0.0001) difference was that the *Gp6* transcript level decreased in vaccinated mice between days 4 and 8 *p.i.*, whereas, concomitantly, it strongly increased in unvaccinated mice.

CLEC2, encoded by *Clec1b*, is a C-type lectin-like receptor on the surface of megakaryo- and thrombocytes known to serve as a receptor for adherence to the endogenous ligand podoplanin (PDPN) [66,68,69]. In the liver of both vaccinated and unvaccinated mice, *Clec1b* was expressed at a relatively high level of approximately 86 above normalization and was responsive to both malaria and vaccination (Figure 5). Upon infection of unvaccinated mice with blood-stage malaria, however, this relatively high constitutive mRNA level in the liver declined to 55 on day 1 *p.i.* before it sharply increased to about 102 on day 4 *p.i.*, reaching its maximal expression of 119 at peak parasitaemia on day 8 *p.i.* Protective vaccination significantly (*p*-value < 0.05) accelerated both the malaria-induced decrease in *Clec1b* expression to approximately 42 on day 1 *p.i.* and, in particular, accelerated the subsequent increase to 110 on day 4 *p.i.*, which was significantly higher (*p*-value < 0.01) than the corresponding level observed in unvaccinated mice. Maximum expression of approximately 119 occurred at peak parasitaemia on day 8 *p.i.*, which was identical to that in unvaccinated mice. This mRNA level of *Clec1b* was maintained until reaching the end of the crisis phase on day 11 *p.i.* and was significantly higher (*p*-value < 0.001) than the corresponding level in unvaccinated mice. At present, it cannot be excluded that the *Clec1b*-encoded CLEC2 was also expressed at very early stages of megakaryocyte development, as, e.g., by megakaryocyte-biased HSCs, since CLEC-2 signaling is known to regulate maintenance of HSCs in the bone marrow [70].

Currently, megakaryocyte-biased HSCs are defined by high density expressions of CD41, high c-KIT expression, or VWF expression [20], whose encoding genes were found here to be expressed at relatively high levels in the liver. Incidentally, VWF is also expressed by endothelial cells [20]. The *Itga2b*-encoded CD41 (integrin alpha 2b, GPIIb, alphaIIb) on the surface of megakaryo-/thrombocytes serves as a receptor for fibronectin, fibrinogen, plasminogen, prothrombin, thrombospondin, and vitronectin [20]. In the liver of unvaccinated mice, *Itga2b* was constitutively expressed at approximately 34 above normalization level on day 0 *p.i.* (Figure 5). Upon infection with blood-stage malaria, the *Itga2b* transcript levels increased and reached their maximum levels of approximately 57 at peak parasitaemia on day 8 *p.i.* Protective vaccination did not significantly affect *Itga2b* expression in response to blood-stage malaria, neither the constitutive expression nor the increase in malaria-induced expression in comparison with unvaccinated mice. *Kit* encodes c-KIT on the surface of HSCs as a marker for self-renewal capability and megakaryocyte bias [20,71,72]. In the liver of both vaccination-protected and unvaccinated mice, both *Kit* and *Vwf* were constitutively expressed at relatively high levels of 53 and 93, respectively, and both genes were responsive to both blood-stage malaria and vaccination (Figure 5). *Kit* expression in the liver of vaccinated and unvaccinated mice differed in response to malaria. In unvaccinated mice, there was almost a steady increase in *Kit* expression, reaching a maximum of approximately 69 at peak parasitaemia. In vaccination-protected mice, however, there was a significant (*p*-value < 0.05) decrease in *Kit* expression at early prepatency on day 1 *p.i.*, before expression significantly (*p*-value < 0.05) increased to approximately 62 on day 4 *p.i.*, which was higher (*p*-value < 0.05) than in unvaccinated mice. *Vwf* expression in response to malaria and vaccination was different from that of *Kit*. In unvaccinated mice, the response of *Vwf* expression to malaria appeared to be delayed during the prepatency phase and began to steadily increase from approximately 90 on day 4 *p.i.* to 150 towards the end of the crisis phase on day 11 *p.i.* In vaccination-protected mice, however, there was initially a significant (*p*-value < 0.05) decrease of *Vwf* expression, which was thereafter highly significantly (*p*-value < 0.01) increased from approximately 80 on day 1 *p.i.* to 105 on day 4 *p.i.*, reaching maximal expression of 145 at peak parasitaemia on day 8 *p.i.*

### 3.5. Vaccination Alters the Malaria-Responsive Expression of Genes also Involved in Megakaryocyte Development

**Figure 6:**
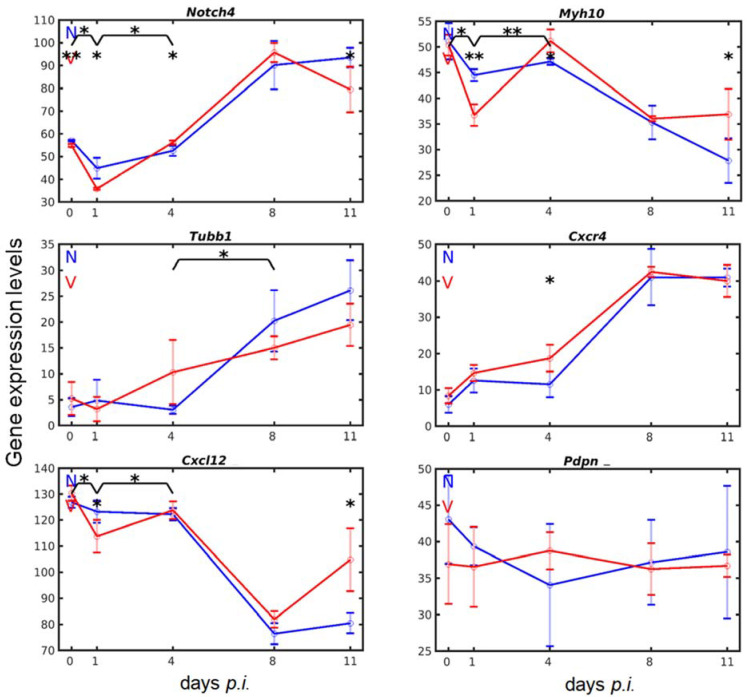
Time series of expressions of genes, known to be involved in megakaryo-/thrombopoiesis, in the liver of vaccination-protected (V, red) and non-vaccinated mice (N, blue). RNA was isolated from individual livers prepared from mice infected with *P. chabaudi* blood-stage malaria on different days *p.i.* Gene expression levels in linear scale are plotted as means of three microarrays ± SD. The number of ‘*’ marks over interval lines between sampling points and over the sampling points indicate statistical significance between two intervals and between a given sampling point, respectively, between vaccinated and non-vaccinated mice: * *p* < 0.05; ** *p* < 0.01.

In the bone marrow, megakaryopoiesis is known to critically involve NOTCH4 signaling at different stages of development [73], TUBB1 (tubulin beta1) for cytoskeleton reorganization, and MYH10 (myosin heavy chain) for terminal maturation processes of megakaryocytes [20]. In the liver, *Myh10*, *Tubb1*, and *Notch4* were constitutively expressed and their expression responded to both blood-stage malaria and vaccination (Figure 6). The constitutive expression level of *Notch4* was approximately 58 above normalization in unvaccinated mice. This level decreased to approximately 45 on day 1 *p.i.*, then slightly increased to approximately 52 on day 4 *p.i.*, before a sharp increase to approximately 90 on day 8 *p.i.* followed by another slight increase to approximately 95 on day 11 *p.i.* Vaccination-protected mice exhibited about the same constitutive level of *Notch4* transcripts as unvaccinated mice, which decreased to approximately 35 upon infection on day 1 *p.i.*, i.e., to a significantly (*p*-value < 0.05) lower level than in unvaccinated mice. Then, *Notch4* transcripts steadily increased, reaching a maximum of approximately 95 at peak parasitaemia on day 8 *p.i.*, before dropping to approximately 80 on day 11 *p.i.*, which was significantly (*p*-value < 0.05) lower than in the liver of unvaccinated mice. The constitutive expression of *Myh10*, approximately 51, resembled that of *Notch4*, but its response to malaria and vaccination was totally different from that of *Notch4*. Infection with malaria of unvaccinated mice decreased expression of *Myh10* to approximately 44 on day 1 *p.i.*, which remained at this level until early patency on day 4 *p.i.*, before a linear decrease reaching a minimum of 28 towards the end of the crisis phase on day 11 *p.i.* In vaccinated mice, however, protective vaccination did not affect the constitutive *Myh10* expression, but blood-stage malaria induced a still larger, highly significant (*p*-value < 0.01) decrease of *Myh10* expression on day 1 *p.i.* than in unvaccinated mice, before *Myh10* transcripts highly significantly (*p*-value < 0.01) increased on day 4 *p.i.* Thereafter, these transcripts declined, reaching about the same level as that found in the liver of unvaccinated mice at peak parasitaemia on day 8 *p.i.* About the same level of *Myh10* mRNA was also found in vaccinated mice on day 11 *p.i.*, which was significantly (*p*-value < 0.05) higher than in unvaccinated mice. *Tubb1* expression differed from *Myh10* and *Notch4*. In particular, the constitutive expression of *Tubb1* in the liver of unvaccinated mice and vaccinated mice was very low compared with *Myh10* and *Notch4*. Infection with blood-stage malaria significantly affected the amount of *Tubb1* transcripts until day 4 *p.i.* Thereafter, however, an almost steady increase in *Tubb1* mRNA occurred, reaching a maximum towards the end of the crisis phase on day 11 *p.i.* In vaccinated mice, however, the time course of *Tubb1* expression in response to malaria appeared to be only delayed for 1 day, before the *Tubb1* mRNA continuously increased towards the end of the crisis phase, but at a significantly lower rate than in unvaccinated mice.

Extension and release of proplatelets from megakaryocytes into the vascular space also requires interaction of the receptor CXCR4 on bone marrow megakaryocytes with its ligand chemokine CXCL12 [20]. In the liver, both encoding genes were constitutively expressed: *Cxcr4* at a very low level of 5 and *Cxcl12* at a very high level of 126 above normalization level (Figure 6). Their responses to blood-stage malaria in both vaccinated and unvaccinated mice occurred inversely with increasing expression of *Cxcr4* and decreasing expression of *Cxcl12* (Figure 6). More specifically, the *Cxcl12* expression in the liver was only minimally affected—if at all—during the prepatency phase by malaria. At the early patency phase on day 4 *p.i.*, however, there began a strong decrease of *Cxcl12* transcripts from 124 to a level of 78 at peak parasitaemia on day 8 *p.i.*, and this level maintained toward the end of the crisis phase on day 11 *p.i.* By contrast, the liver of vaccinated mice responded to blood-stage malaria with a significantly (*p*-value < 0.05) larger decrease in *Cxcl12* expression during the first day of infection, before expression significantly (*p*-value < 0.05) increased to that level of unvaccinated mice on day 4 *p.i.* Thereafter, expression also strongly decreased until peak parasitaemia, as in unvaccinated mice, before it turned to a significant (*p*-value < 0.05) increase in vaccination-protected mice towards the end of the crisis phase on day 11 *p.i. Cxcr4* expression increased in response to malaria very similarly in vaccinated and non-vaccinated mice. Only on day 4 *p.i.*, there was a significantly (*p*-value < 0.05) higher expression in vaccinated mice than in unvaccinated mice.

Megakaryocyte growth and proplatelet formation were also strongly promoted by interactions of CLEC-2 on the surface of megakaryocytes and thrombocytes with its endogenous natural ligand podoplanin (PDPN) mainly expressed in the vasculature [74]. In the liver, *Clec1b* and *Pdpn* were constitutively expressed without any significant difference between vaccination-protected and unvaccinated mice. In response to malaria, however, *Clec1b* expression strongly differed between vaccinated and unvaccinated mice, while expression of *Pdpn* was not significantly affected, neither by malaria nor by vaccination (Figure 6).

### 3.6. Vaccination Enhances Transcripts of Exportable Proteins in Liver-Localized Cells of the Megakaryocyte Lineage

**Figure 7:**
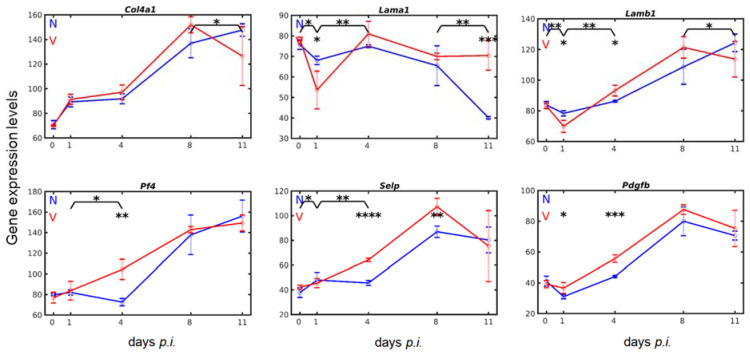
Hepatic expressions of genes known to be expressed by megakaryo-/thrombocytes. RNA was isolated from individual livers prepared from vaccination-protected (V, red) and non-vaccinated mice (N, blue) infected with *P. chabaudi* blood-stage malaria on days 0, 1, 4, 8, and 11 *p.i.* Gene expression levels in linear scale are plotted as means of three microarrays ± SD. The number of ‘*’ marks over interval lines between sampling points and over the sampling points indicate statistical significance between two intervals and between a given sampling point, respectively, between vaccinated and non-vaccinated mice: * *p* < 0.05; ** *p* < 0.01; *** *p* < 0.001; **** *p* < 0.0001.

In the bone marrow, megakaryocytes and their progenitor cells are obviously capable of manipulating their niche microenvironments, for example, by producing the type IV collagen COL4A1 and the laminins LAMA1 and LAMB1 [75]. In the liver, *Col4a1* was constitutively expressed at about the same level of 70 above normalization in both vaccination-protected and unvaccinated mice (Figure 7). Additionally, the response of expression to blood-stage malaria was about the same in both vaccinated and unvaccinated mice. There was an increase in expression from about 70 to about 90 above normalization level on day 1 *p.i.* and expression remained at this level until day 4 *p.i.*, before the maximum expression of 135 was reached at peak parasitaemia on day 8 *p.i.* The only difference occurred towards the end of crisis, when expression significantly (*p*-value < 0.05) declined in the liver of vaccination-protected mice in comparison with unvaccinated mice. Incidentally, the time course of *Col4a1* expression resembled that of *Runx1*.

The constitutive expression of *Lama1* was approximately 77 and that of *Lamb1* was approximately 83 above normalization level. Their responses to malaria differed in both vaccination-protected and unvaccinated mice. *Lama1* expression in the liver of unvaccinated mice was initially impaired until early patency on day *4 p.i.* before it declined, reaching its minimum of approximately 40 towards the end of the crisis phase (Figure 7). In vaccinated mice, however, there was a significantly (*p*-value < 0.05) higher initial decrease in expression from 78 to 54 at early prepatency on day 1 *p.i.* than in unvaccinated mice, which was followed by a highly significant (*p*-value < 0.01) increase to a maximum of approximately 81 on day 4 *p.i.*, before the expression decreased to approximately 72 at peak parasitaemia on day 8 *p.i.* and remained at this level towards the end of the crisis phase, which was significantly (*p*-value < 0.001) higher than in unvaccinated mice. The response of *Lamb1* expression to malaria differed from that of *Lama1*, though there was an almost similar unresponsive phase in unvaccinated mice until day 4 *p.i.*, as also observed for *Lama1* (Figure 7). Thereafter, *Lamb1* expression linearly increased to a maximum of approximately 126 towards the end of crisis on day 11 *p.i.* In vaccination-protected mice, *Lamb1* transcripts initially decreased on day 1 *p.i.*, followed by a highly significant (*p*-value < 0.001) increase on day 4 *p.i.* This linear increase continued to reach its maximum at peak parasitaemia, before *Lamb1* transcripts significantly (*p*-value < 0.05) declined towards the end of the crisis phase on day 11 *p.i.*

Megakaryocytes and thrombocytes are known to produce and release PF4 (platelet factor 4), PDGFb (platelet-derived growth factor b), and SELP (P-selectin). In the liver of adult mice, *Pf4*, *Selp*, and *Pdgfb* were constitutively expressed: the *Pf4* transcripts were most abundant at a level of approximately of 80 above normalization level, whereas *Selp* and *Pdgfb* were expressed at a level of about 40 (Figure 7). The expression of these three genes in response to blood-stage malaria differed in unvaccinated mice and vaccinated mice. Common to unvaccinated mice was a relatively low response of expression to blood-stage malaria until early patency on day 4 *p.i.*, before a highly significantly increased expression at peak parasitaemia on day 8 *p.i.* Compared with unvaccinated mice, the liver of vaccinated mice responded to malaria at early patency on day 4 *p.i.* with a significantly higher expression, though at different levels of significance for *Pf4* (*p*-value < 0.01), *Selp* (*p*-value < 0.0001), and *Pdgfb* (*p*-value < 0.001). The highest expression levels of *Pf4* and *Pdgfb* were reached at peak parasitaemia on day 8 *p.i.*, and were not significantly different from those in unvaccinated mice. Only *Selp* was significantly (*p*-value < 0.01) higher expressed at peak parasitaemia in vaccinated mice than in unvaccinated mice.

### 3.7. Validation of Microarray Results by Quantitative RT-PCR

**Figure 8:**
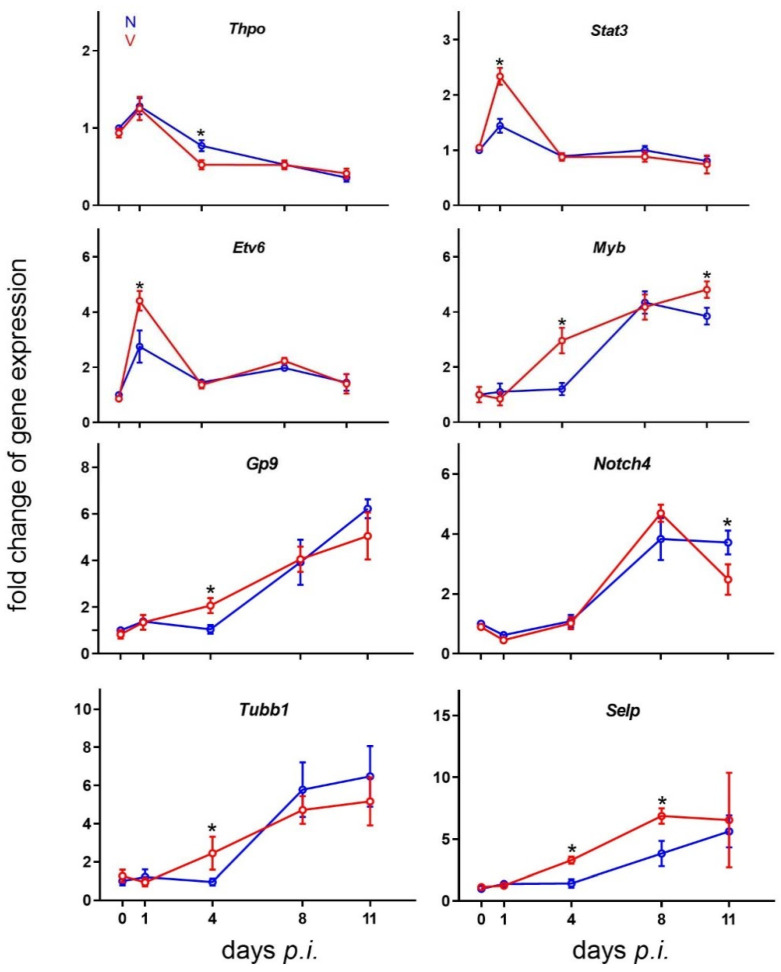
Quantitative real-time PCR of mRNAs in the liver of vaccinated and non-vaccinated Balb/c mice. RNA was isolated from individual livers prepared from mice infected with *P. chabaudi* on different days *p.i.* Means of duplicate determinations, performed with liver aliquots from three different mice, with SD. ‘*’ indicate significant differences between vaccinated and non-vaccinated mice (*p* < 0.05).

Protective vaccination was found by microarrays to significantly reshape hepatic expression of genes in cells related to the megakaryocyte lineage in response to primary blood-stage infections of *P. chabaudi* malaria (Figure 3, Figure 4, Figure 5, Figure 6 and Figure 7). To independently re-examine these microarray data, the arbitrarily selected eight genes *Thpo*, *Stat3*, *Etv6*, *Myb*, *Gp9*, *Notch4*, *Tubb1*, and *Selp* were subjected to quantitative RT-PCR analysis. Figure 8 shows the RT-PCR results of the time courses of gene expressions plotted as -fold changes expressed mRNA, whereas the microarray results indicate mRNA levels above the normalization level. Conspicuously, the expressions of these eight genes in response to malaria took time courses in both vaccinated and non-vaccinated mice, which largely resembled those determined by microarrays. For instance, *Thpo*, *Myb*, *Gp9*, *Tubb1*, and *Selp* had higher expressions on day 4 *p.i.* in vaccinated mice than in unvaccinated mice, and also *Stat3* and *Etv6* on day 1 *p.i.*, *Selp* on day 8 *p.i.*, and *Myb* on day 11 *p.i.*, while *Notch4* was lower expressed on day 11 *p.i.*

## 4. Discussion

This study provides evidence that the liver of female Balb/c mice responds to primary infections of blood-stage malaria of *P. chabaudi* with the expression of genes, generally known to be preferentially expressed by cells of the megakaryocytic lineage in the bone marrow. Most remarkably, this gene response to malaria occurred significantly faster in the liver of vaccination-protected mice than in unvaccinated mice, evidenced at early patency on day 4 *p.i.*, when the liver exhibited significantly higher levels of malaria-induced transcripts of the genes *Selp* and *Pdgfb* at *p*-values < 0.0001, *Gp5* at *p*-value < 0.001, and *Mpl*, *Gp1ba*, *Gp1bb*, *Gp6*, *Gp9*, *Pf4*, and *Clec1b* at *p*-values < 0.01. Transcripts of these genes are known to occur in thrombocytes, either encoding MPL, GPIba, GPIbb, GPV, GPIX, GPVI, and CLEC2 on their surface or the exportable proteins SELP, PDGFb, and PF4. Thrombocytes, in particular PF4, were recently shown to act as effective killers for erythrocytes infected with different human *Plasmodium* species [39] and SELP as a host receptor for *P. falciparum* merozoite surface protein 7 (PfMSP7) [76], whose deletion impairs parasite invasion into host erythrocytes [77]. A possible explanation of the vaccination-accelerated increase in malaria-responsive transcripts on day 4 *p.i.* might be an increased immigration of peripheral thrombocytes, or specific subpopulations of thrombocytes [78], into the liver to support local defense mechanisms against blood-stage malaria, not only to eliminate *Plasmodium*-infected erythrocytes [38,39,79], but also to contribute to local healing processes of malaria-induced acute injuries [80].

It is also plausible that the vaccination-accelerated increase in malaria-responsive transcripts on day 4 *p.i.* does not only reflect an increased immigration of peripheral thrombocytes into the liver, but rather an increased production of thrombocytes generated from increased liver-intrinsic megakaryocytes. Indeed, all the identified transcripts are known to occur not only in thrombocytes, but also in megakaryocytes. For instance, *Mpl* is known to be expressed as TPO receptor on all cells of the megakaryocyte lineage including megakaryocyte-biased HSCs [56,81]. The heteromeric surface membrane complex GPIB-GPV-GPIX is known to be expressed during terminal maturation and polyploidization and during cytoskeletal rearrangement and proplatelet production of megakaryocytes [20,21]. The surface collagen receptor GPVI and the *Clec1b*-encoded surface protein CLEC2 are also expressed on megakaryocytic cells and are known to serve as heme and hemin receptors to activate thrombocytes [66,67,82], which in turn can induce macrophage extracellular traps [83], possibly also involved in eliminating *Plasmodium*-parasitized erythrocytes in the liver.

In contrast to *Gp6* and the *Gp* genes encoding the GPIB-GPV-GPIX surface complex, *Clec1b* revealed a dramatic decrease in expression upon malaria infection during early prepatency, before a strong increase from day 1 *p.i.* to early patency on day 4 *p.i.* Both the initial down- and the subsequent up-regulation in response to malaria were significantly larger in vaccinated mice than in unvaccinated mice, presumably without any malaria-responsive involvement of the endogenous CLEC2-ligand PDPN [74], since *Pdpn* expression was not significantly affected, neither by blood-stage malaria nor by protective vaccination. Moreover, the liver of vaccination-protected mice responds to blood-stage malaria with a similar early down- and up-regulation of the expression of the genes *Myh10* (*p*-value < 0.01), *Lama1* (*p*-value < 0.05), *Lamb1* (*p*-value < 0.05), *Notch4* (*p*-value < 0.05), *Cxcl12* (*p*-value < 0.05), *Kit* (*p*-value < 0.05), and *Vwf* (*p*-value < 0.05). The RUNX-activated or -repressed MYH10 regulates megakaryocyte polyploidization [20,84]; NOTCH4 signaling is required for development of earlier progenitors and NOTCH4 downregulation for terminal megakaryocyte commitment [20]; the megakaryocyte-produced laminins LAMA1 and LAMB1 create an appropriate bone marrow microenvironment for megakaryocytes [20,75]; *Kit* and *Vwf*, besides *Itga2b*, can be very early expressed by megakaryocyte-biased HSCs [20,72]; CXCL12 regulates maintenance of HSCs in the bone marrow [75], as is also known for CLEC-2 signaling [70]. The early downregulation of transcripts, in particular that of *Clec1b*, may therefore signal a reprogramming in the very early pathway of megakaryocyte development, possibly also associated with megakaryocyte fragmentation and/or release of microparticles [28], rather than with a possible emigration of megakaryocytic cells out of the liver. Consequently, our data would suggest that extramedullary development of megakaryocytes and thrombocytes, respectively, may occur in the liver in response to blood-stage malaria, delayed in non-vaccinated mice and accelerated in vaccination-protected mice. Vaccination obviously accelerates the liver-intrinsic megakaryo-/thrombopoiesis.

In the bone marrow, megakaryopoiesis is currently envisaged as to be also critically regulated at the transcriptional level, which requires different transcription factors to interact in different combination as activators and/or repressors, which are encoded by genes, as e.g., *Stat3*, *Etv6*, *Runx1*, *Zfpm1*, *Nfe2*, *Fli1*, *Gata2*, and *Myb* among others [20,57,62]. Our data indicate that, also in the liver, there apparently exists an intricate network of transcription factor-encoding genes, whose expression is significantly up- or downregulated in response to blood-stage malaria in vaccination-protected mice. For instance, protective vaccination induced an early increase in expression of *Stat3*, *Etv6*, and *Runx1* at early prepatency on day 1 *p.i.* and, concomitantly, an early decrease in *Stat5*, *Zfpm1*, *Nfe2*, *Fli1*, and *Myb*, which was followed by highly significant increases in expression of *Runx1*, *Fli1*, and *Myb* at early patency on day 4 *p.i.*, before *Runx1* and *Gata2* were significantly downregulated towards the end of the crisis phase on day 11 *p.i.* However, only two of these genes, namely *Runx1* and *Fli1*, are currently envisaged as to be involved exclusively in megakaryopoiesis, whereas *Myb* is currently debated as to be exclusively expressed in cells of the erythrocyte lineage [20,57,58]. In the liver of vaccination-protected mice, *Runx1*, *Fli1* and *Myb* display the most significant changes in their expression patterns in comparison with unvaccinated mice. In particular, the time courses of *Runx1* expression are very similar to those of *Col4a1* and *Cxcr4*. Thus, it is plausible that at least *Runx1* and *Fli1* may also play a critical role in the regulation of liver-intrinsic megakaryopoiesis, whereas *Myb* may be exclusively involved in the regulation of extramedullary erythroblastosis in the liver [17]. On the other hand, *Myb* expression in the liver of vaccination-protected and unvaccinated mice takes a very similar time course as those of *Gp1ba*, *Gp1bb*, *Gp5*, and *Gp9* encoding membrane surface proteins on megakaryocytes and thrombocytes in the liver. This suggests that *Myb* may not only be involved in the regulation of liver-intrinsic erythroblastosis—if at all—but also in liver-intrinsic megakaryopoiesis, both accelerated by protective vaccination.

Vaccination-accelerated liver-intrinsic megakaryo-/thrombopoiesis may be explained to be due to increased immigration of megakaryocyte progenitors from the bone marrow and/or from the lung into the liver. Indeed, megakaryocytes exist not only in the bone marrow but also in the lung [26,85,86]. Lung megakaryocytes originally derived from the bone marrow [86] were reported to emigrate from the lung and even constitute the HSC compartment in the bone marrow [26]. However, a more attractive, though speculative, interpretation of our data is that liver-intrinsic megakaryo-/thrombopoiesis may proceed from megakaryocyte-biased HSCs populations localized in the adult liver, which may be even able to bypass faster the canonical pathway of megakaryopoiesis in vaccination-protected mice than in unvaccinated mice. Recently, it became known that not only the fetal liver, but also the adult liver contains fetal liver-derived HSCs, and these HSCs are capable of generating liver-resident type 1 innate lymphoid cells (ILC1s) [87].

Furthermore, our data showed an inverse relationship between *Thpo* and *Mpl* transcripts during liver-intrinsic megakaryo-/thrombopoiesis, evidenced particularly between day 1 and 4 *p.i.*, when the significantly higher initial *Thpo* expression decreased and, inversely, the significantly lower initial *Mpl* expression increased. Both decline and increase, respectively, occurred significantly faster in vaccinated mice than in unvaccinated mice. Recently, evidence was provided that hepatic TPO production is driven predominantly by GPIba [88,89,90]. Our data showed that *Gp1ba* expression during liver-intrinsic megakaryo-/thrombopoiesis takes about the same time course as *Mpl* expression, just as the expressions of *Gp1bb*, *Gp5*, and *Gp9*, all together encoding the membrane surface complex GPIB-GPIV-GPIX on megakaryocytes and thrombocytes. It is therefore possible that GPIba becomes increasingly ineffective to stimulate hepatic production of TPO with progressing infections of blood-stage malaria and/or the vaccination-accelerated liver-intrinsic megakaryo-/thrombopoiesis in response to blood-stage malaria becomes increasingly less sensitive to hepatic TPO. The latter view may be in accordance with findings showing that, in the bone marrow, later stages of megakaryopoiesis, as e.g., formation and release of proplatelets, become increasingly TPO-independent and even TPO-dispensable [20,91,92].

## 5. Conclusions

Our data provide further evidence for the view that the liver is an important organ of the host defense against blood-stage malaria and presumably promotes the efficacy of protective vaccination. Indeed, vaccination accelerates liver-intrinsic megakaryo-/thrombopoiesis that may contribute to survival of otherwise lethal blood-stage malaria of *P. chabaudi.*

## Data Availability

Microarray data are available at the NCBI’s GEO database with accession number GSE129133.
